# Diagnosing Colour Vision Deficiencies Using Eye Movements (Without Dedicated Eye-Tracking Hardware)

**DOI:** 10.3390/jemr18050051

**Published:** 2025-10-02

**Authors:** Aryaman Taore, Gabriel Lobo, Philip R. K. Turnbull, Steven C. Dakin

**Affiliations:** 1 School of Optometry & Vision Science, The University of Auckland, Auckland 1142, New Zealand; glob765@aucklanduni.ac.nz (G.L.); p.turnbull@auckland.ac.nz (P.R.K.T.); s.dakin@auckland.ac.nz (S.C.D.); 2 New Zealand National Eye Centre, The University of Auckland, Auckland 1142, New Zealand; 3 UCL Institute of Ophthalmology, University College London, London EC1V 9EL, UK

**Keywords:** optokinetic nystagmus, colour vision, eye tracking

## Abstract

Purpose: To investigate the efficacy of a novel test for diagnosing colour vision deficiencies using reflexive eye movements measured using an unmodified tablet. Methods: This study followed a cross-sectional design, where thirty-three participants aged between 17 and 65 years were recruited. The participant group comprised 23 controls, 8 deuteranopes, and 2 protanopes. An anomaloscope was employed to determine the colour vision status of these participants. The study methodology involved using an Apple iPad Pro’s built-in eye-tracking capabilities to record eye movements in response to coloured patterns drifting on the screen. Through an automated analysis of these movements, the researchers estimated individuals’ red–green equiluminant point and their equivalent luminance contrast. Results: Estimates of the red–green equiluminant point and the equivalent luminance contrast were used to classify participants’ colour vision status with a sensitivity rate of 90.0% and a specificity rate of 91.30%. Conclusions: The novel colour vision test administered using an unmodified tablet was found to be effective in diagnosing colour vision deficiencies and has the potential to be a practical and cost-effective alternative to traditional methods. Translation Relevance: The test’s objectivity, its straightforward implementation on a standard tablet, and its minimal requirement for patient cooperation, all contribute to the wider accessibility of colour vision diagnosis. This is particularly advantageous for demographics like children who might be challenging to engage, but for whom early detection is of paramount importance.

## 1. Introduction

Around 4% of people—8% of males and 0.5% of females—have a congenital colour vision deficiency (CVd) [1], the most common forms being protanomaly or deuteranomaly (red or green colour deficiencies, respectively). Such conditions lead to poor discrimination between colours falling along the protan and deutan confusion lines—directions in the chromaticity space that represent the red–green colour confusion axes for individuals with L- or M-cone deficiencies, respectively [2]. This impaired discrimination impacts an individual’s ability to perform everyday tasks (such as driving, preparing food, etc.) and can influence career choices [3]. Consequently, a colour vision assessment is an important part of a comprehensive eye examination. Such an assessment usually relies on Ishihara plates, which are suitable only for screening (i.e., indicating that there may be a problem with colour vision) as they provide neither the severity nor the type of colour vision deficiency [4]. The anomaloscope [5] is widely considered the gold-standard instrument for distinguishing cases of congenital red–green colour vision deficiency. However, when we refer to the anomaloscope as a “gold standard,” we do so in this narrow context only. It is not a comprehensive tool for all aspects of colour vision assessments: it does not reliably quantify the severity of the red–green loss, performs poorly in the presence of acquired blue–yellow deficiencies, and is sensitive to individual differences in the optical densities of L and M cones [6]. Moreover, this specialised piece of equipment is uncommon in clinics, as it requires a trained operator and extended periods of attention from the patient. It is also particularly challenging for children, in whom the detection of colour vision deficiencies is most valuable.

**Figure 1:**
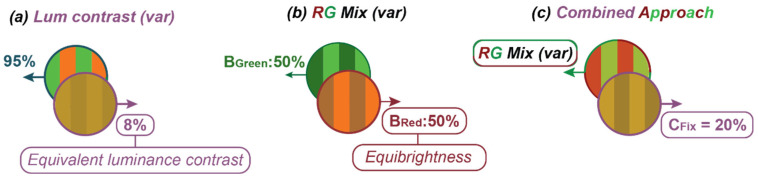
A motion-nulling paradigm to quantify the contribution of colour to motion. (**a**) The contrast of a luminance-defined grating required to “null” the motion of a colour-defined grating (i.e., to induce a percept of flicker) is known as equivalent luminance contrast (*C*_Eq_). (**b**) A similar nulling approach quantifies the contrast at which red and green appear equally bright (equi-brightness). (**c**) By modifying the relative red–green contrast in the coloured drifting pattern, in the presence of a (fixed-contrast) luminance-defined grating drifting in the opposite direction, we can simultaneously determine both equi-brightness and *C*_Eq_.

To overcome the limitations related to operator expertise, prolonged patient engagement, and poor suitability for paediatric testing, we have described a simple-to-use, rapid, and objective measure of functional colour vision based on eye movements made in response to motion [7]. Our test exploits a link between the subjective impression of the colour and eye movements made in response to the motion of a coloured stimulus [8]. Figure 1a illustrates the approach, which follows the work of Anstis & Cavanagh [8] and uses a stimulus comprising two superimposed sine-wave gratings moving in opposite directions. One “colour-defined” component is made up of inter-digitated red and green drifting gratings; the other “luminance-defined” component is a simple yellow grating with the luminance of the sum of two superimposed red and green gratings. When presented with this stimulus, the viewers’ eye movements are consistent with their subjective impression of the “dominant” direction of motion, and no coherent eye movements arise when the two components appear to be of equal contrast. In this subjective equivalence case, the stimulus appears to flicker, and the individual gratings’ motion is said to have been “nulled”. The minimum contrast of the luminance-defined grating required to null the motion of a colour-defined grating (Figure 1a) quantifies the contribution of the colour to motion. This contribution—termed the equivalent luminance contrast (*C*_Eq_)—is around 8% in participants with typical colour vision (CVn), but is much lower in patients with CVds [7,8].

While the *C*_Eq_ could be used to classify CVn from CVd, in order to distinguish protanomaly from deutranomaly we also need to determine when red and green appears equally bright (Figure 1b). To determine this, one could again use a nulling paradigm where superimposed red and green gratings (of various relative contrasts) moved in opposite directions (Figure 1b) [9]. In this case, a CVn participant should experience equi-brightness at an equal ratio of red to green (i.e., *B*_red_ = 50%), whereas a CVd participant would need their defective colour to be a higher physical contrast to achieve equi-brightness (e.g., *B*_red_ typically greater than 60% for protanopes). However, rather than running two separate tests (i.e., Figure 1a,b) we followed Anstis & Cavanagh [8] by fixing the contrast of the yellow nulling grating and varying the R/G balance of the coloured grating (Figure 1c) to simultaneously determine the *C*_Eq_ and the red–green equi-brightness point. The two measures derived from this test—the red/green equi-brightness level and the *C*_Eq_—were used to determine the participants’ colour vision status.

We measured an eye movement response comprising both involuntary optokinetic nystagmus (OKN) [10] and the voluntary/active tracking of the stimulus (also known as smooth pursuit) [11]. OKN is a type of eye movement made reflexively to large-field visual motion and consists of a sequence of periods of slow-tracking eye movements in the direction of the stimulus interspersed with rapid corrective saccades in the opposite direction. Such eye movements are mediated by partially shared brainstem and spinal pathways [12] and serve to minimise retinal slip [13]. We refer to the combined eye movement response as directed eye movements (DEMs), which we define as any stimulus-aligned tracking movement—including OKN and smooth pursuit. Participants were instructed to follow the stimulus when it felt natural to do so. To quantify the strength of this tracking response, we calculated the ratio of the mean gaze velocity to the mean stimulus velocity, a measure we refer to as the DEM gain.

Our previous report [7] described how this approach led to the >90% accurate categorisation of CVd. Although the system used relied on a consumer-grade eye tracker costing around USD 200, the requirement for such hardware is still a barrier to the adoption of this test in clinics. Further, although the test ran on a consumer laptop, we had to carefully luminance-calibrate the built-in display to ensure colour accuracy. Here we set out to determine if we could run our test on an unmodified consumer tablet (an Apple iPad Pro) using “software-only” eye tracking (using the visible light output of the built-in front-facing webcam). To determine the impact of an uncalibrated display, we measured the spectral characteristics of a variety of different models of iPads and then modelled the impact of varying screen characteristics on our results.

We report that the software-only version of our test running on the iPad classifies people by their colour vision status almost as well as our previous system that used a dedicated eye tracker. As a result, we are now able to provide a rapid, fully automated, and objective assessment of functional colour vision to clinicians through a software download onto their unmodified iPad device. By lowering barriers in this way, we hope to improve colour vision testing not only in optometry practice but also in other settings where reliable colour vision testing is essential, e.g., in the development of gene replacement therapies for congenital achromatopsia, a genetic condition characterised by impaired colour discrimination along all three axes of colour vision corresponding to the three cone classes.

## 2. Methods

### 2.1. Participants

We recruited 33 participants (17 females, 16 males, 17–65 years), of which 23 were controls with normal colour vision, 8 were deuteranopes (all male), and 2 were protanopes (both male). CVd status was determined using an anomaloscope. The protocols and procedure complied with the Declaration of Helsinki, and informed consent was obtained from all participants prior to the experiment.

### 2.2. Apparatus

A Type-I Neitz anomaloscope (Model OT-II) was used to perform a standard diagnosis of red–green CVd [14]. For the eye-tracking tasks, we presented stimuli and recorded eye movements using an iPad tablet computer (2019 iPad pro 12.9”, LCD display, 120 Hz, 2732 px by 2048 px). The screen was viewed under standard room lighting (Illuminant D65) and at approximately 40 cm without head/chin support. The luminance of RGB components of the screen was linearised in software using measurements made with a Konica Minolta LS-110 photometer. Stimuli were created in MATLAB R2023a (The MathWorks, Natick, MA, USA) using elements of the PsychToolbox [15]. The front camera of the iPad recorded a series of visible light images of the user at 60 Hz to estimate the orientation of each eye relative to the face in the horizontal and vertical direction (referred to as leftEyeTransform and rightEyeTransform in the ARkit framework, Apple’s augmented reality software platform that provides real-time face and eye tracking on iOS devices). The experiment was performed without any chin or headrest, although participants were instructed to attempt to maintain a constant head position.

### 2.3. Stimuli

Similarly to our previous work [7], stimuli were superimposed pairs of vertical sine-wave gratings moving in opposite horizontal directions (Figure 1c and Video S1). Gratings had a peak spatial frequency of 0.5 cycles per degree, moved at 8 deg/sec, and were subtended 46.54° by 27.26°. Participants were presented with two 90s long movies, each containing a fixed luminance contrast of either 10% or 20%. Based on our previous study, these conditions lead to data that best separate between participants with different colour vision statuses. Each movie comprised forty 2.5s trials. Direction of grating (left or right) and the proportion of red (25%, 37.5%, 50%, 62.5%, 75%) in the red–green component was randomised across trials to minimise the build-up of optokinetic aftereffects, with each combination of direction and proportion in red of the coloured grating being repeated four times. Participants were instructed to allow their eyes to follow the stimulus when it felt natural to do so. Eye movements were scored by taking the ratio of the mean tracking velocity to the mean stimulus velocity—a measure we refer to as *DEM Gain*. To classify eye movements as either saccades or tracking movements, we adopted the same approach used in our previous works [7,16]. In brief, we calculated an eye velocity threshold, which was used to classify instantaneous estimates of horizontal eye velocity as either saccadic or tracking movements. The threshold was set so to maximise the distance travelled by the eye, assuming DEM in the stimulus direction.

### 2.4. Analysis

**Figure 2:**
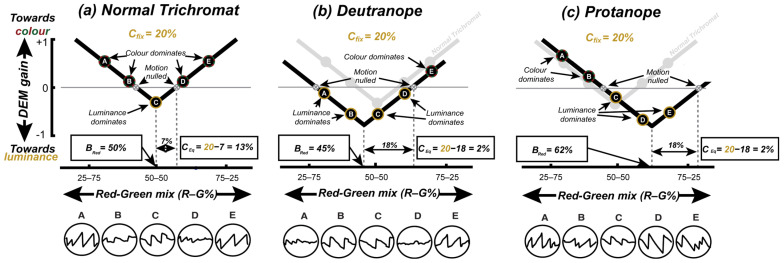
Schematic plots of the strength (gain) of directed eye movement (DEM) for (**a**) a trichromat, (**b**) a deuteranope, and (**c**) a protanope viewing variable-contrast colour gratings in the presence of a fixed-contrast (20%) luminance grating (as shown in Figure 1c). Plotting the direction/strength of eye movements as a function of red–green mixture leads to “V” functions, where the horizontal shift captures when red and green appear equally bright (*B*_red_), and vertical shift quantifies equivalent luminance contrast (*C*_Eq_).

Plotting DEM gain against the red–green luminance balance (Figure 2a–c) yields V-shaped functions which have their minima at the equi-brightness point and typically cross the zero-gain line at two points (the two red–green mixtures leading to motion nulling). We fit these data using a V-function with three free parameters (Equation (1)):

(1) $$R=\;S\left|M_{\mathrm{red}}\;-\;B_{\mathrm{red}}\right|\;+\;A$$

where *R* is the predicted DEM response, and *M*_red_ is the red component of the red–green colour mixture of the coloured grating. The three fit parameters are *B*_red_ (the red–green mix that minimises *R*, i.e., the equi-brightness point), and *A* and *S* are offset and scaling parameters, respectively. *C*_Eq_ is then defined as the difference between the fixed luminance contrast *C*_fix_ and the distance of the nulls from *B*_red_.

Figure 2a–c are representative plots of DEM strength versus red–green colour balance for CVn and CVd. Note that the DEM strength in Figure 2a–c is characterised as positive or negative for whether the tracking phase of DEM was consistent with the direction of the colour or luminance component, respectively.

For a typical CVn observer (Figure 2a), the V-function is relatively symmetrical around the physical equi-luminance point (50–50 on the x-axes). The luminance contrast leading to nulling of colour motion (*C*_Eq_ = 7%) is lower than the fixed luminance (20%), to yield an equivalent luminance contrast of 20 − 7 = 13%. For a CVd observer (Figure 2b), responses are dominated by the luminance-defined grating, shifting the “V” downwards and reducing *C*_Eq_ to only 2%. A more severe CVd (Figure 2c) also leads to a horizontal shift in equi-brightness (and the whole V-function) towards the defective colour.

Note that for CVn (Figure 2a) points A and E show a strong positive response (in the direction of the coloured grating) when the colour mix is dominated by either red or green. In contrast, for CVd the endpoints of the V-function are shifted towards the zero line, as colour contributes less to their motion response (*C*_Eq_ = 2% for the deuteranope and protanope). The black outlined circles below the plots show representative DEM responses for points A-E (horizontal eye displacement plot against time). Note that for CVn (Figure 2a) points A and E show strong saw tooth patterns towards the right and strong saw tooth patterns towards the left when close to equiluminance (point C).

## 3. Results

**Figure 3:**
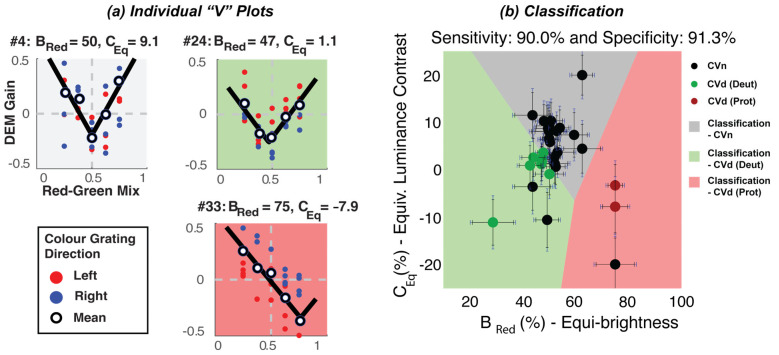
Differences in measures of directed eye movements (DEMs) between normal trichromats and colour vision deficient observers. (**a**) DEM gain (categorised for direction of the colour component) measured with a 20% fixed luminance contrast. Plots in the white, green, and red panels are from a trichromat, deuteranope, and protanope individual, respectively. Red and blue symbols show DEM gain from trials when the coloured grating moved left or right, respectively, with the mean gain across trials indicated by open symbols. Note the downward shifts in gain measures from colour vision deficient observers (red and green panels), indicating that their percept is dominated by the luminance component of stimuli. (**b**) Equi-brightness plot against equivalent luminance contrast for all thirty-three participants. Boundaries of the coloured regions were derived using a K-means algorithm that sought to best separate the three groups. Error rates indicate the percentage of misclassified participants.

Figure 3a shows the pattern of responses across three participants (with different colour vision statuses) observing stimuli moving with a fixed luminance contrast of 20%. Plots in the white, green, and red panel show data from a CVn, deuteranope, and protanope individual, respectively. Red and blue symbols show gain estimates from trials when the coloured grating moved left or right, respectively, with the mean gain across trials indicated by open symbols. Note that while the protanope (red panel) has a V-function shifted to the right, the deuteranope (green panel) has a V-function that is almost symmetrical around the physical equiluminance (similar to the CVn). As such, while *B*_red_ best separated protanopes from CVn, *C*_Eq_ best separated deuteranopes from CVn.

Using both the *B*_red_ and *C*_Eq_ we sought to classify these participants into their respective colour vision groups. However, if you recall, we ran this test twice—once for a low-fixed and once for a high-fixed luminance contrast (10% and 20%) condition. As such, we received two “V” fits and therefore two estimates of *B*_red_ and *C*_Eq_. Like our previous work [7], we opted to select parameters from the “deeper” V-function (quantified using the magnitude of the scaling parameter) of the two *C*_fix_ levels. This was decided because the visual inspection of our data suggested that some parameters from some conditions were unreliable, usually as a result of noisy DEM responses that were fit to “shallow” V-functions. A scatterplot of individual estimates of the equi-brightness against the equivalent luminance derived in this way is shown in Figure 3b. The selected “V” fits were bootstrapped (using 1000 resamples) to derive confidence intervals on estimates of *B*_red_ and *C*_Eq_—denoted by the black error bars in Figure 3b. During bootstrapping, estimates of *B*_red_ were clipped in the range of 25–75 and *C*_E_ in the range from −20 to 20 to avoid estimates made from degenerate bootstrapped data sets. Also, note the blue error bars, which denote the confidence intervals on the *B*_red_ and *C*_Eq_ for varying iPad displays; these are elaborated later on in *Discussion*.

An unsupervised machine learning algorithm (K-means clustering) [17] was then used to partition participants into three clusters, in which each participant belonged to the cluster with the nearest mean (Figure 3b). The cluster centre values for each group were as follows: the centre for CVn was located at *B*_red_ = 51 and *C*_Eq_ = 7; the centre for CVd (deut) was comparable, with a *B*_red_ = 44, but this was further skewed towards the higher physical contrast of their defective colour (green), and a lower *C*_Eq_ = −2; and finally, the centre for CVd (proto) was characterised by an extremely skewed *B*_red_ = 75 and *C*_Eq_ = −10. A visualisation of the cluster results is presented in Figure 3b. K-means requires an initial guess of the “centroid” (i.e., the centre point) for each cluster before the iterative optimisation begins. Based on previous findings [8], the initial centroid positions were set to *B*_red_ = (40, 50, 62.5) and *C*_Eq_ = (0, *C*_fix_, 0), respectively. Our test indicated a high sensitivity (90.0%) and specificity rate (91.30%), against the diagnosis made using the gold-standard anomaloscope.

Looking at the data, we note error bars with a size of ±4.3% for *B*_red_ and ±4.16% for *C*_Eq_ on average across participants; this is comparable to the error bars generated from the data from our previous work [7], which used a commercial-grade eye tracker (± 4.95% for *B*_red_ and ± 4.35% for *C*_Eq_). This suggests that despite the iPad’s limited eye-tracking capability, its noisier gaze estimates did not significantly influence the relative OKN responses measured for each stimulus level. Rather, the similar variance between devices indicates significant noise in the OKN responses elicited by participants for each stimulus level. Noise in the OKN responses of participants is also noted in our previous work. We attribute this to the random switching participants exhibited between tracking the colour and luminance components under conditions where both provided coherent motion cues. However, at or near points where the chromatic and luminance gratings cancelled each other’s motion signal, participants may have perceived flickers rather than coherent motions, making tracking challenging during those trials. More generally, we conclude that, in an effort to continuously track, participants were more or less inclined to engage in the random attentional tracking of either the colour or luminance component at times.

We also note the CVn individual is classified as a CVd individual (proto), as a result of his or her extremely high *B*_red_ and low *C*_Eq_ estimates. We have noted overlaps in the *B*_red_ and *C*_Eq_ estimates of CVn and CVd (deut) in our previous work [7] but not with CVn and CVd (proto). Looking at this participant’s response, we note an average OKN gain of <0.05 across all five stimulus levels. The almost insignificant OKN response even for the high-contrast conditions (i.e., 25/75 and 75/25 Red–Green Mix) suggests that the participant may have had difficulty engaging in the task. While the cause of the poor OKN response is unknown, in a real-world setting, such cases would be automatically flagged using a simple gain threshold (e.g., average DEM gain < 0.1), prompting a re-test. Because our system runs in real time, it would also be feasible to detect poor tracking performances during the task itself and provide corrective feedback or reminders to follow the stimulus.

## 4. Discussion

A key motivation for developing this test was to progress accessible and accurate screening. While our previous work [7] proved the feasibility of our paradigm, the use of a commercial-grade eye tracker (to measure OKN) made it challenging to deploy as an affordable at-home or in-clinic screening tool. Here we showed the successful use of an iPad to (a) present the stimulus and (b) track participant eyes in response to the stimulus using the front-facing camera. The sensitivity (90.3%) and specificity (91.3%) rate achieved from the iPad against the gold-standard anomaloscope was almost identical to our initial test that made use of a commercial-grade eye tracker (sensitivity = 90.9% and specificity = 91.30%). These performance metrics, however, reflect the specific sample used in this study; future work involving a larger and more representative distribution of colour vision deficiencies—including milder cases—will be important for establishing generalisable sensitivity and specificity.

Consistent with our previous findings, a subset of normal trichromats were misclassified in the current study. In our earlier work, we showed that these misclassifications were not due to deficits in colour vision but instead reflected variability in participants’ optokinetic responses. Specifically, we identified a form of “response bias”, where DEM responses fluctuated between tracking the chromatic component of the stimulus and residual luminance signals. This likely occurred because participants were instructed to “follow the stimulus”, and when motion cues were ambiguous, some appeared to switch arbitrarily between components.

A natural question is why this response bias emerged in our paradigm but not in the study by Anstis and Cavanagh [8], which showed a stronger separation between colour-deficient and colour-normal observers. A key difference lies in the task structure: Anstis and Cavanagh [8] used a method-of-adjustment procedure to null the perceived motion, whereas our approach used a two-alternative forced choice (2AFC) design based on the DEM. In our previous study, we showed that this response bias also appears in subjective keyboard-based motion reports, indicating that the effect is not specific to eye movement measurements. Instead, it appears to stem from the forced-choice format itself, which likely introduces variability by requiring a response even when the motion percept is ambiguous.

Our stimulus design employed a large-field display (47° × 27°) to elicit strong OKN responses. While this approach is advantageous for triggering OKN responses, it also introduces potential variability in chromatic processing across the visual field. Peripheral chromatic sensitivity is known to decline with eccentricity, and the ratio of L to M cones can vary substantially between individuals [18]. These factors may contribute to classification errors. Future work may benefit from systematically manipulating the stimulus size.

Compared to the anomaloscope, our test was significantly shorter (~2 min vs. 20 min) and much simpler to administer, making it fit for use on both young and old participants who are generally unable to comply, and it is able to be performed by less skilled operators.

Existing studies have used the iPad and its built-in eye-tracking framework (ARKit) to record eye movements during reading [19]. However, this is the first study that we are aware of that uses ARKit to measure OKN. Unlike traditional eye trackers that rely on infra-red images, the ARKit framework on the iPad processes visible light images to estimate gaze [20]. The limited accuracy of the iPad (~3.18 deg) [20] generally makes it difficult to use in contexts that rely on estimating the absolute gaze position on a screen. However, in our pilot testing we noted that the iPad provides adequate linear interpolation from the physical eye movement (in degrees) to the iPad’s numerical representation of the eye movement (−1 to 1). This makes it fit to measure the OKN strength, which is more reliant on relative and not absolute gaze positions. This also means we did not need to carry out any eye tracking calibration procedure, further reducing the test time and overcoming compliance issues.

Another significant challenge for a wide-scale roll-out of our initial iteration of the test was to do with the display calibration. Our initial test used a standard display monitor, which had considerable variation in display consistency across different monitors of the same model. This meant in-clinic or home displays would need to be calibrated on site, typically requiring specialised equipment (i.e., photometer) to match the luminance of the red and green channels. However, the previous literature suggests that the iPad display can provide adequate colour reproduction errors between different devices of the same model [21]. In practice, this assumes that users are operating the device under standardised settings—for example, with True Tone and Night Shift disabled and the brightness set to maximum. However, key parameters such as the screen brightness can be programmatically detected within the app, and participants can be prompted to adjust their settings if they fall outside acceptable ranges. As such, at-home and or in-clinic testing may be possible by way of supported iPad models whose displays have been tested in house. This would allow for universal screening on any “out-of-the-box” supported iPad.

To test for the impact of the display variation on our procedure, we measured the hypothetical shift in estimates of the *B*_red_ and *C*_Eq_ across 30 iPads—#10 iPad Airs, #10 iPad Airs 2, #6 iPad Pros (12.9 inch) (2nd Gen), #2 iPad Pros (11 inch) (2nd Gen), and #2 iPads (7th Gen). We first began by measuring the radiant power (the energy emitted in Watts) of each iPad display using a tele-spectroradiometer (Photo Research PR-655 SpectraScan Spectroradiometer). For each channel (red and green) we measured the radiant power across the visible light spectrum (wavelengths 380 to 700 nm) at five intensity levels (0, 64, 128, 191, 255), while keeping all other channels (including the blue channel) at intensity level 0. The individual measures were photometrically weighted, then integrated to give a measure of the total luminous flux (in lumens) at the five intensity levels for the red and green channels. An inverted gamma correction function was then calculated for each channel to linearise the luminance, and a scaling ratio was used to match the luminance of the linearised red and green channels. Across displays we calculated an average value of γ = 2.2 (0.040) for the red channel, γ = 2.19 (0.034) for the green channel, and a ratio of 0.33 (0.020). Using the average γ_red_, γ_green_, and ratio as set display configurations for all the iPad displays, we calculated hypothetical measures of the *B*_red_ and *C*_Eq_ at *C*_fix_ = 20% for a fictional observer with no colour vision—to capture the device’s objective equi-luminance and motion null points solely based on the luminance interaction. We noted a standard deviation of 1.61% for the *B*_red_ and a negligible standard deviation of 0.61% for the *C*_Eq_ across displays. These standard deviations were used to derive the confidence intervals in Figure 3b (in blue). These standard deviations were identical across all simulated observer types—including protanopes, deuteranopes, and trichromats—because the display-level shifts affect all observers in the same way, independent of their colour vision status. Note how minimally the display impacts the estimates when compared to the general confidence intervals (in black).

Our work suggests that our iPad-based test could be used by clinicians and patients alike, without the need for screen or eye tracker calibrations. Our test is a simple, reliable, and automated colour vision assessment that could provide an accurate and in-depth colour vision diagnosis in clinics and or homes.

## Data Availability

The data presented in this study are available on request from the corresponding author. The data are not publicly available due to participant confidentiality requirements.

## Supplementary Materials

The following supporting information can be downloaded at https://www.mdpi.com/article/10.3390/jemr18050051/s1, Video S1: This article contains a video as additional online-only material. The following should appear online only: Video S1.

## Abbreviations and Acronyms

CVd (colour vision deficient), CVn (typical colour vision), Deut (deuteranope), DEM (directed eye movement), OKN (optokinetic nystagmus), and Proto (protanope).
